# Mitigating Container Damage and Enhancing Operational Efficiency in Global Containerisation

**DOI:** 10.3390/s25072019

**Published:** 2025-03-24

**Authors:** Sergej Jakovlev, Tomas Eglynas, Mindaugas Jusis, Valdas Jankunas, Miroslav Voznak

**Affiliations:** 1 Department of Telecommunications, VSB-Technical University of Ostrava, 17. listopadu 2172/15, 708 00 Ostrava-Poruba, Czech Republic; miroslav.voznak@vsb.cz; 2 Marine Research Institute, Klaipeda University, Universiteto al. 17, Klaipėda, 92295 Klaipėda, Lithuania; tomas.eglynas@ku.lt (T.E.); mindaugas.jusis@ku.lt (M.J.); valdas.jankunas@ku.lt (V.J.)

**Keywords:** impact detection methodology, acceleration, vibration, handling process, transportation

## Abstract

**Highlights:**

**What are the main findings?**

- We introduce the Impact Detection Methodology (IDM), a system to monitor and detect crane-induced impacts on containers in real time, demonstrating effectiveness in mitigating handling-related damage.
- We identify key factors contributing to container damage, such as unsuccessful hooking attempts during above-deck lifts due to spreader oscillations and high operational workloads.

**What is the implication of the main findings?**

- Integrating the IDM with crane management technologies and sway control systems can enhance operational precision, reduce container damage, and improve port efficiency.
- Broader real-time monitoring and advanced analytics adoption can support sustainable growth in global containerisation by reducing operational costs and mitigating financial risks.

**Abstract:**

The global containerisation industry, while significantly advancing international trade, faces persistent challenges related to infrastructure capacity, environmental impact, and operational efficiency. One critical yet under-researched issue is the physical damage that containers endure during handling operations, particularly at port terminals. This paper examines the complexities of container handling, focusing on damage caused by quay crane activities, especially during corner hooking. Such damage compromises container integrity, impacts cargo safety, and increases operational costs. To address these concerns, we present the Impact Detection Methodology (IDM), a system designed to monitor and detect impacts in real time, enhancing operational precision and safety. Preliminary studies conducted at Klaipeda City port demonstrate the IDM’s effectiveness, though limited data have constrained validation. Our research underscores the need for broader experimentation to confirm the IDM’s potential in mitigating container damage. Key findings indicate that unsuccessful hooking attempts predominantly occur when containers are lifted from above-deck positions, influenced by spreader oscillations and high operational workloads. This paper also highlights the importance of integrating sway control systems with existing crane management technologies to assist operators in reducing handling errors. Enhanced monitoring and data analysis are essential for improving container handling processes, supporting sustainable growth in global containerisation, and mitigating financial risks.

## 1. Introduction

The expansion of global containerisation has reshaped logistics, but the industry’s rapid growth has led to mounting challenges that vary by region [1,2]. The global handling volume of containers reached over 811 million TEUs in 2021, a dramatic increase from the 619 million TEUs handled just a decade prior, as reported by the 2021 United Nations Conference on Trade and Development [3]. Despite its achievements, the industry faces serious issues in infrastructure capacity, environmental impact, and operational efficiency, with each global region experiencing unique hurdles [4,5,6]. As international trade intensifies, each major region grapples with unique challenges in container logistics [7], leading to congestion, capacity issues, and increased operational costs. For instance, in Europe, ports are struggling with congestion and capacity issues [8,9], particularly in Northern European hubs like Rotterdam, Hamburg, and Antwerp, which handle nearly 40% of the region’s container volume. Increased container demand has exacerbated bottlenecks, especially as post-pandemic cargo surges continue to disrupt port operations [10], often leading to cargo backlogs and impacting regional supply chains [11,12]. Europe is now focusing on digitalisation and port automation [13,14,15] as part of the “Europe Fit for the Digital Age” initiative to streamline port efficiency, while the EU is imposing stricter environmental regulations on shipping to curb emissions [16]. Likewise, in North America, container volumes have surged strongly from pre-pandemic levels, and U.S. West Coast ports like Los Angeles (Container Volume Statistics 2021 [17]) have seen dwell times increase during peak periods in 2021, registering record-breaking container volumes in 2021, processing over 10 million TEUs. The increased congestion has driven shipping rates, which tripled during the pandemic, reflecting the broader strain on North American logistics networks [18]. On the other hand, in South America, containerisation is hampered by limited infrastructure and uneven access to major ports. According to ECLAC [19], the region’s port performance lags significantly, with Brazil, Argentina, and Chile facing capacity constraints and bottlenecks at key ports [20]. This often results in lengthy and higher logistics costs, typically higher than those in developed regions. South American countries are working on enhancing intermodal connectivity and expanding port infrastructure, yet financial constraints and political instability can delay progress. Going to Asia, especially China, Japan, and South Korea, remain at the forefront of container traffic, with ports like Shanghai, Singapore, and Busan ranking among the busiest globally [21,22]. Shanghai alone handled over 40 million TEUs in 2021 [23], underscoring the massive scale of containerisation in Asia [24,25,26].

However, China’s strict COVID-19 measures caused shutdowns, leading to regional bottlenecks [27], which rippled globally [28]. The strain on container availability has been aggravated by an imbalance in trade flows, as containers from Asia to Europe and the Americas are often returned empty, resulting in billions in annual inefficiencies across the sector. This imbalance affects cost structures and pressures Asian shipping companies to explore container-sharing and leasing options. These regional challenges are also compounded by growing environmental concerns [29,30], as container ships contribute significantly to global carbon emissions [31,32,33,34], highlighting the urgent need for sustainable modernisation across the containerisation industry. Environmental concerns are prevalent across all retainer ships, which account for around 3% of global carbon emissions, and the International Maritime Organization (IMO [35]) warns that maritime emissions could increase by 50% by 2050 without significant intervention. Asia and Europe are adopting alternative fuels and stricter emission standards, but these initiatives are still in the early stages and face high transition costs. North America is gradually introducing LNG-powered vessels and other greener solutions, yet adoption rates remain slow. These distinct challenges across Europe, the Americas, and Asia underscore the global containerisation sector’s need for full and innovative modernisation. As each region adapts to its constraints [36], addressing the main pressing issues of congestion, environmental impact, and efficiency remains central to the industry’s evolution, yet not the only thing.

While the industry grapples with environmental challenges [7] and the need for sustainable and smart practices, an equally pressing yet often overlooked issue is the physical damage containers endure, directly impacting cargo safety, supply chain efficiency, and operational costs. Global containerisation has fuelled growth in international trade [37,38]. Still, damage to shipping containers is an under-researched issue that poses significant risks to cargo safety, supply chain efficiency, and operational costs. As containers travel through diverse and often challenging environments, they are exposed to rough handling, extreme weather, and other stresses compromising their structural integrity [39]. Despite the staggering number of TEUs handled annually globally, the focus on damage prevention, monitoring, and maintenance remains limited [40]. Damaged containers can lead to cargo loss, delays, and higher insurance premiums, resulting in significant financial implications for the shipping industry and its clients. Container damage is surprisingly frequent [41]. According to a report by the World Shipping Council [42], approximately 1582 containers are lost at sea each year, often due to structural failures exacerbated by heavy seas or container stack collapses. Yet, despite these numbers, monitoring and maintenance remain secondary considerations, largely due to cost concerns, a lack of standardised monitoring protocols, and proper research in this area.

The risk of container damage is especially critical as global trade expands. Between 2010 and 2021, the volume of containerised cargo grew by nearly 31%, stressing already overburdened infrastructure and increasing the likelihood of damaged goods. In North America and Europe, port congestion has led to hasty loading and unloading, increasing the chances of handling-related damage. For instance, the Port of Los Angeles, one of the busiest in the world, reported a 25% increase in container volume from pre-pandemic levels, amplifying pressures on both equipment and workforce [17]. In Asia, where ports like Shanghai handle upwards of 47 million TEUs annually, this same growth has led to even greater congestion [43], resulting in higher risks of container misalignment, improper stacking, and accelerated wear on containers. Monitoring container conditions throughout transport could prevent or mitigate many of these issues, yet standardised approaches are lacking.

**Figure 1:**
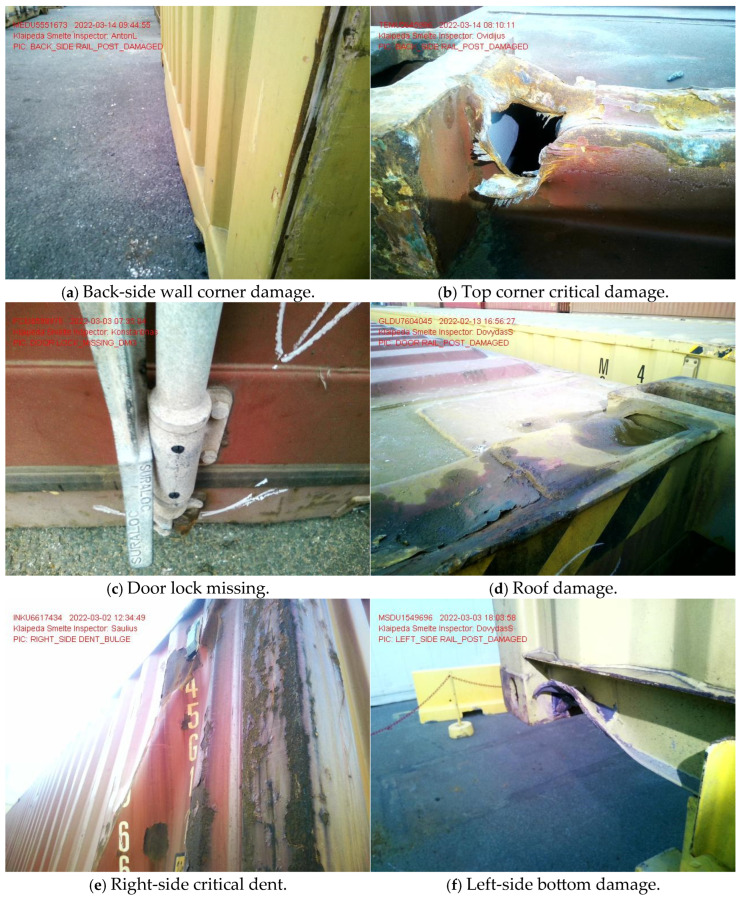
True examples of damages that were detected by the operators of Klaipeda Port (examples taken from LKAB “Klaipedos Smelte” container terminal operations database).

Most shipping companies rely on visual inspections and intermittent checks (see Figure 1 for several examples from the visual inspection at the LKAB “Klaipėdos Smeltė” container terminal), which are insufficient for tracking gradual structural deterioration. Various existing methods, such as vision-based detection [44,45,46], GPS tracking [47,48,49], and other sensor-based techniques [50,51,52,53], offer alternative approaches for monitoring container conditions. Vision-based systems, while effective for external structural assessments, often struggle in environments with low visibility, occlusions, or adverse lighting conditions, making them less reliable for real-time impact detection during crane operations. GPS tracking provides valuable insights into container location and movement patterns but lacks the granularity required to detect handling-induced impacts at the operational level. Other sensor-based methods, such as acoustic or strain gauge systems, require extensive infrastructure modifications, making them less practical for widespread implementation, especially in smaller terminals.

**Figure 2:**
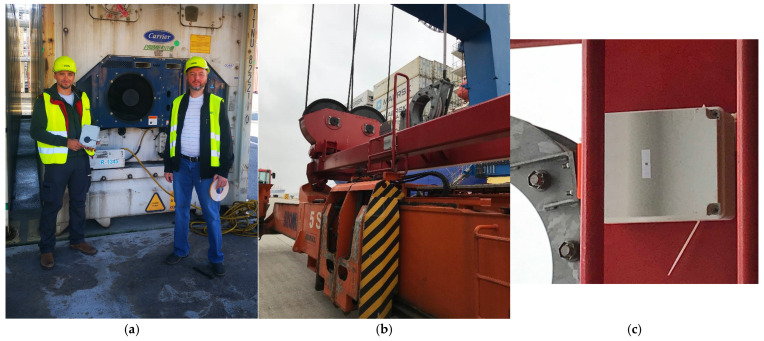
Research team members Dr. S. Jakovlev (check the left side of (**a**)) and Dr. V. Jankūnas (check the side of (**a**)) at the LKAB “Klaipėdos Smeltė” container terminal in Klaipėda city conducting experimental studies with smart sensors and the integrated Impact Detection Methodology (IDM), with the acceleration sensor mounted on the spreader of the quay crane shown in its intended position for impact detection (**b**), and a close-up view of the sensor mounting point on the spreader structure (**c**) [60,61].

The IDM approach offers a distinct advantage by directly detecting and analysing impact forces during handling, allowing for real-time identification of improper container hooking and potential structural damage while using only a small number of measurement units. Unlike vision-based or GPS methods, the IDM is designed to function effectively in dynamic port environments without reliance on external visual conditions or extensive pre-installed tracking infrastructure. Furthermore, by employing acceleration and vibration sensors with optimised signal processing techniques, the IDM provides a high degree of accuracy in detecting impacts that could compromise container integrity. Implementing such technologies and performing critical experimental research on-site (see Figure 2) would help extend the service life of containers and reduce the frequency and severity of damages, but costs and logistics concerns have slowed adoption.

We strongly point out that the need for more comprehensive research and investment into container condition monitoring and maintenance is critical to sustaining the growth of global containerisation. Yet, the issue we examined previously remains relatively ignored compared to other areas of logistics optimisation [15,54,55,56], such as automation [11,57,58] and digitalisation [59], not to mention the economical estimates. As global trade volumes are expected to continue rising, effective and autonomous container inspection is becoming increasingly essential to avoid further costs and inefficiencies. The financial impact of container damage on global trade is substantial.

Each incident of container damage can lead to cascading delays, as damaged containers must be removed, inspected, and sometimes replaced, slowing down logistics chains. This delay can exacerbate congestion in already overwhelmed ports; for instance, delays at European ports are frequently prolonged due to damaged or improperly handled containers. The costs of these delays ripple through supply chains, driving up shipping costs, which have already seen a drastic increase in some regions during peak congestion periods. Damaged containers may also result in rejected or destroyed cargo, particularly for industries shipping sensitive or perishable goods, adding to the financial losses. Moreover, the lack of consistent monitoring and predictive maintenance systems for container conditions exacerbates these challenges [62]. Considering these circumstances, our team is at the forefront of research in this field, leading among our academic peers. This work builds upon prior research efforts to underscore the challenges and provide statistically validated data, demonstrating that such monitoring activities are essential and can be effectively conducted using modern techniques, systems, and standards. Later in this paper, we will present evidence-based metrics and statistics to help practitioners working with these processes better understand the situation and assess the significance of such monitoring activities.

## 2. Materials and Methods

**Figure 3:**
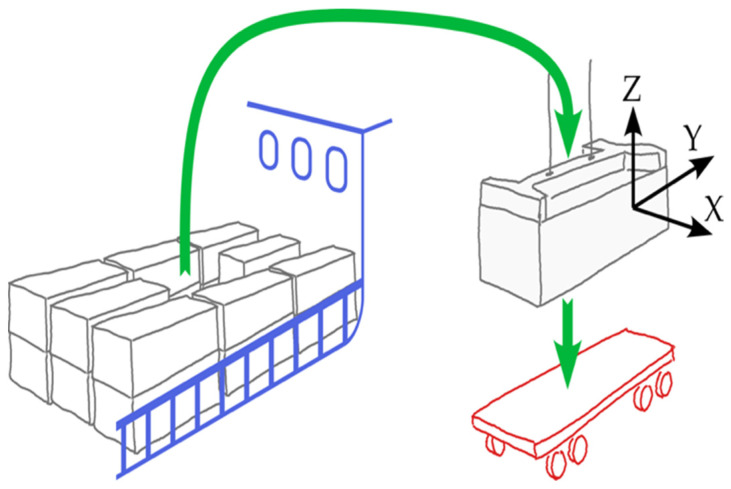
Container handling process description (shown as the green arrows for ship-to-shore handling operation).

Our research investigates the complexities of container handling operations, concentrating on container damage caused by quay crane activities, particularly during corner hooking. This damage impacts container integrity, safety, and efficiency. We previously presented the Impact Detection Methodology (IDM) at Klaipeda City port, demonstrating its real-time impact detection capabilities, including the sensors and overall technical means, but in rather limited handling operations, which constrained its validation. This study highlights the necessity for broader experimentation and deeper analysis to confirm the IDM’s effectiveness in mitigating container damage. Initial findings indicate that unsuccessful hooking often occurs when containers are lifted from above-deck positions (see Figure 3), where controlling spreader oscillations is more challenging due to fast-paced operations and high workloads.

Expert insights indicate that current procedures may contribute to these issues, necessitating further review. We also observed that containers near the top of a stack are routinely unloaded according to daily plans, suggesting standardised handling methods, despite a high rate of acceptable faulty operations. Improving spreader stabilisation and examining sway control systems for integration with existing crane management systems could assist in addressing these challenges.

To briefly demonstrate the logic behind the IDM framework, it utilises a signal processing approach in which acceleration and vibration data undergo preprocessing to eliminate high-frequency noise and isolate relevant impact events. This process employs statistical thresholding techniques to distinguish between normal operational vibrations and unintended impact forces. The threshold values are derived from empirical data and adjusted dynamically according to handling conditions, ensuring adaptability to varying operational scenarios. While the current study focuses on validating the IDM’s effectiveness within a controlled port environment, our future research will extend the methodology to different operating conditions, further refining parameter optimisation for diverse use cases. We have utilised the following measurement equipment to acquire the data:

- The model was “Steval–Mki109V3”.
- The data acquisition rate was 100 Hz.
- We worked in low-power mode.
- The acceleration detection was ±16 g.

**Figure 4:**
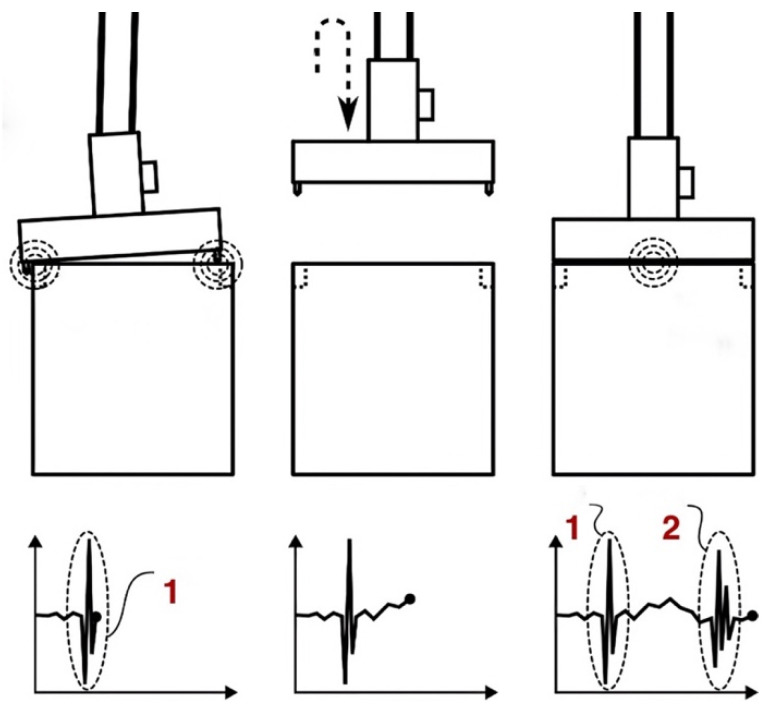
IDM visual explanation: 1—initial impact to the container; 2—secondary impact to the container (in the graphs, horizontal axes are time, and vertical axes are acceleration).

The Impact Detection Methodology (IDM) detects repeated impacts within a specific loading cycle. By analysing data and evaluating the characteristics of the loading process, the IDM identifies the container hooking phase. During this phase, the IDM employs filtering methods (as detailed in previous studies) to detect deviations from standard handling and impacts between the spreader and container. Figure 4 provides a visual representation of this process:

1. When the crane lowers the spreader onto a container for lifting, an impact occurs as the spreader meets the container.
2. If properly aligned, the spreader’s four hooks match the container’s slots, creating a slight and expected vibration on contact—a normal and acceptable impact.
3. This controlled impact allows the hooks to secure the container, ensuring a safe lift without further impact on non-reinforced areas.
4. However, operator error or external factors can cause the hooks to strike non-reinforced areas, resulting in unacceptable impacts and potential container damage.
5. Such misalignment disrupts the loading process, risks structural damage, and extends loading times due to the need for re-hooking attempts.

This figure illustrates three key stages in the container handling process. In the first stage (left), the spreader descends improperly onto the container due to operator error or external factors, resulting in unsuccessful hooking. This necessitates the operator to lift and reposition the spreader for another attempt (middle).

**Figure 5:**
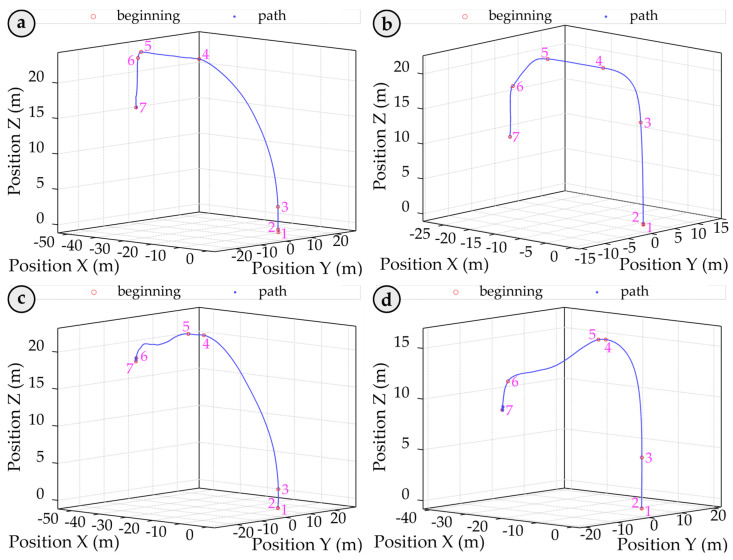
Spreader trajectory during a container handling cycle, with key movement phases marked by points 1 to 7 on the blue curve—from detachment on the truck, vertical ascent, horizontal transfer, to descent and container hooking on the ship. The zero Z-axis level indicates the detachment height. (**a**,**b**) Show first-attempt hooking cycles (e.g., cycle 133, 108), while (**c**,**d**) depict cycles requiring a second attempt (e.g., cycle 71, 152), highlighting variations in trajectory due to operator actions.

The second attempt successfully achieves a hook in the final stage (right). The initial failed impact is marked as “1”, while the successful second impact is labelled as “2”, indicating effective hooking on the second try. This underscores the critical importance of precision in container handling and demonstrates the IDM’s role in monitoring these significant events. This study specifically addresses the detection of such events and analyses the associated statistics, including their location, height, and frequency. By accurately identifying and documenting handling errors, this research lays the foundation for future studies to develop strategies to mitigate these issues and enhance operational processes. The IDM for detecting crane spreader impacts on containers involves several steps: filtering the signal, identifying peak values through statistical analysis, setting threshold levels, pinpointing potential impact events based on these thresholds, isolating critical impacts by grouping related events, and identifying repeated impacts within the same area. The threshold detection algorithm within the IDM identifies event frequency, currently at 100%, by establishing the optimal threshold level as the starting point for impact detection. This threshold evaluation technique has proven effective in real-time applications. To enhance clarity, we illustrate various container transportation pathways when containers are moved from the ships onto the trucks using quay cranes (see Figure 5). Figure 5 depicts the average trajectory of the spreader during a handling cycle. At point 1, the spreader detaches from the container on the truck, ascends to its maximum height (point 4), moves horizontally (to point 5), and then descends towards the container aboard the ship (point 7), where the new container is hooked. This tracking provides valuable insights into the dynamics of container handling, presenting opportunities for efficiency improvements and impact reduction. In all graphs, the zero division of the Z coordinate marks the spreader’s position upon detachment from the container on the truck. Parts of Figure 5a,b represent crane spreader trajectories in the ship-to-shore direction during container loading cycles where the operator hooks the container on the first attempt (cycle 133; group (a), cycle 108). Cycles requiring a second attempt are illustrated in graphs in Figure 5c,d (cycle 71; group (a), cycle 152). Noticeable variations in trajectory shapes emphasise the critical role of accurate crane operation and highlight differences in operator success across attempts, underscoring the potential for further operational enhancements.

## 3. Results

Examining the trajectory and accelerations of the container crane spreader can ascertain whether the operator successfully hooked the container on the first attempt.

**Figure 6:**
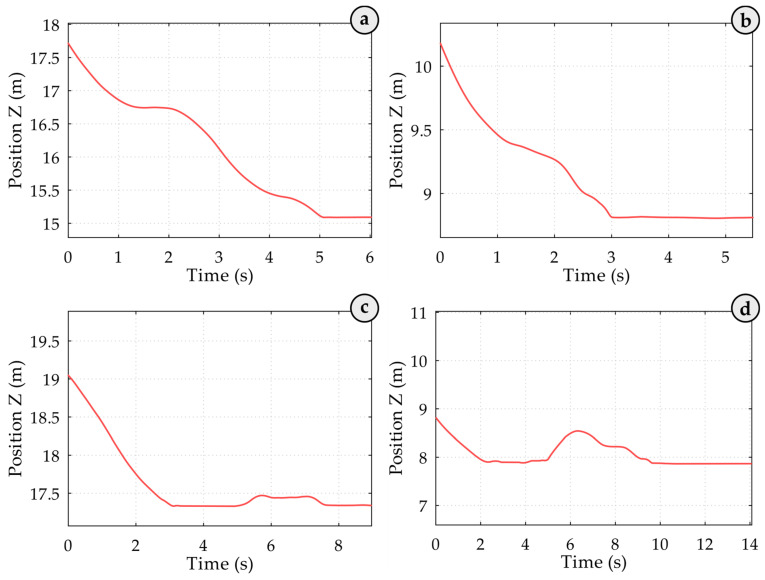
Spreader Z-axis position over time during the final phase of the loading cycle. In (**a**,**b**), the spreader reaches its lowest point at 5.0 s and 3.0 s, respectively, remaining unchanged, indicating successful first-attempt container hooking. In (**c**,**d**), the spreader is briefly lifted at 5.2 s and 5.0 s, followed by noticeable fluctuations caused by sway and repositioning attempts, with successful container impact occurring on the second attempt at 7.5 s and 9.4 s, respectively.

**Figure 7:**
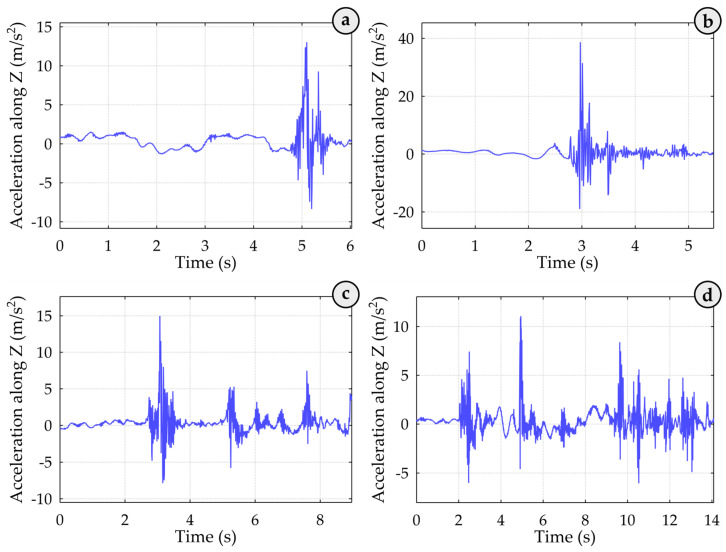
Acceleration of the quay crane spreader during container approach. Here: (**a**,**b**) shows the impact and locking mechanism vibrations during the successful first attempt hooking, and (**c**,**d**) display acceleration fluctuations from spreader sway and repositioning during the second attempt hooking, with a successful impact around 9.4 s, indicating added operational delay and potential container stress.

In the related graphs in Figure 6, the Z coordinate of the spreader is shown over time at the end of the loading cycle. At the 5.0 s (see Figure 6a) and 3.0 s (see Figure 6b) time marks, the spreader reached its lowest point, which remained unchanged until the end of the loading cycle. In the corresponding crane spreader acceleration graphs (see Figure 7a,b), large fluctuations in acceleration values were recorded simultaneously, likely caused by the spreader impacting the container. The container-locking process likely caused the following smaller fluctuations. Based on these acceleration and coordinate graphs, it can be concluded that the container was hooked on the first attempt.

At the 5.2 s (see Figure 6c) and 5.0 s (see Figure 6d) time marks, the operator lifted the spreader slightly. From 5.3 s to 7.5 s (see Figure 6c) and from 4.8 s to 9.4 s (see Figure 6d), the acceleration graph shows significant fluctuations caused by the sway of the incorrectly positioned and subsequently raised crane spreader and the operator’s attempt to reposition the spreader correctly. At the 7.5 s (see Figure 6c) and 9.4 s (see Figure 6d) time marks, the operator lowered the spreader again, and on the second attempt, it impacted the container successfully, based on further measured spreader position data. This impact generated acceleration vibrations (Figure 7c,d), followed by vibrations in the spreader’s body caused by the locking mechanisms. Consequently, not only might the container structure have been damaged, but an additional 4.5 s (7.4 s) were added to the loading process. The trajectory of the spreader shows whether it hooked the container on the first attempt. At approximately the 5.0-s mark in the trajectory graph, the operator slightly lifted the spreader. Between roughly 4.8 and 9.4 s, the acceleration graph shows significant fluctuations, likely due to the sway of the initially misaligned and then raised spreader as the operator worked to reposition it correctly. Around 9.4 s, the operator lowered the spreader again, resulting in a second impact on the container. According to subsequent position data, this placement was successful. This impact produced acceleration vibrations, followed by vibrations from the spreader’s locking mechanisms. Such impacts not only compromise the container’s structural integrity due to the initial faulty hooking but also add a delay to the operation, the time required to achieve a successful hook. Full analysis of the handling process, the entire 210-record dataset showed that the operator successfully hooked the container 30 times on the second attempt and 6 times on the third attempt.

**Figure 8:**
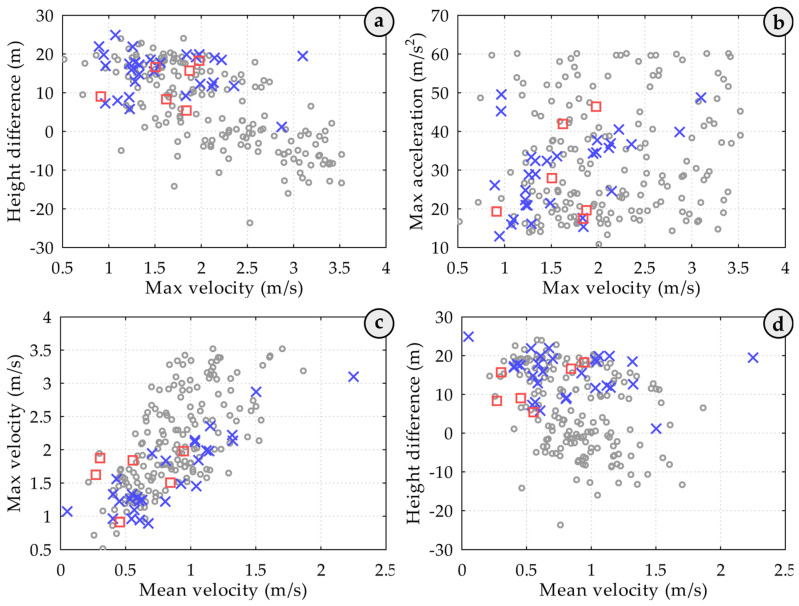
Graphs illustrating the relationships between hooking attempts and various kinematic parameters of the crane spreader during container handling operations. Here: (**a**) shows the relationship between the number of attempts, spreader lowering height difference, and maximum speed; (**b**) shows the relationship between the number of attempts, maximum acceleration, and maximum speed; (**c**) shows the relationship between the number of attempts, maximum speed, and average speed; (**d**) shows the relationship between the number of attempts, spreader lowering height difference, and average lowering speed.

Next, to measure distance and velocities, the time interval was taken for the spreader’s movement from point 1 (hooking of the container on the truck) to point 7 (placement of the crane spreader onto the container on the ship) after the first impact (Figure 8). Figure 8 illustrates the dependence of the number of attempts on the drop height, average speed, and maximum speed.

Figure 8a shows the relationship between the number of attempts, height difference, and maximum speed. For cases where hooking was successful on the first attempt, the Pearson correlation coefficient (PCC) was calculated at −0.70, whereas for cases with two or more hooking attempts, the PCC value was −0.12. This suggests that successful first-attempt hooking is primarily determined by the effective positioning of the spreader above the stack; the greater the lowering distance, the more momentum the operator can build for the crane spreader.

Figure 8b displays the relationship between the number of attempts and the maximum acceleration and speed. For cases where the container was successfully hooked on the first attempt, the calculated PCC value of 0.14 is significantly lower than that of the smaller sample.

Figure 8c depicts the relationship between the number of attempts, maximum speed, and average speed. For successful first attempts, the PCC is 0.67, while for attempts involving at least one repeat, the PCC increases to 0.80. The larger sample size did not significantly alter these findings, indicating a strong linear correlation between maximum and average lowering speeds, likely influenced by the shape of the descent trajectory.

**Figure 9:**
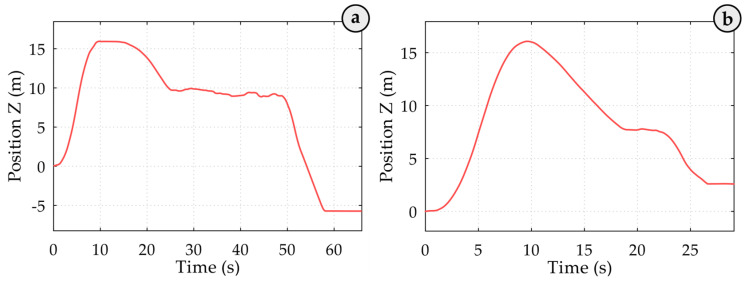
Demonstration of the spreader lowering process into the ship’s internal stack shafts. Here (**a**) shows the spreader trajectory during a loading cycle, demonstrating lifting, repositioning, and descent into the stack, and (**b**) shows the loading cycle with operator-adjusted descent timing, highlighting operational variability and its potential impact on container handling precision.

Figure 8d shows the relationship between the number of attempts, height difference, and average lowering speed of the crane spreader. For successful first attempts, the PCC is −0.37, whereas for repeated attempts, it is −0.03. This indicates that a greater spreader descent distance allows the operator to choose a higher lowering speed, with unsuccessful hooking more probable when containers are positioned higher than the truck. For successful first attempts, the PCC is 0.12, indicating, as with the smaller sample, that there is no linear correlation between the average lowering speed and the impact strength of the spreader on the container. Additionally, repeated hooking attempts often occur at lower lowering heights. This may be attributed to the absence of stack guides above the deck, which help dampen spreader oscillations. Below the ship’s deck, stack guides are in place, necessitating that the operator first make precise adjustments before lowering the crane spreader to that level. The new measurements clearly illustrate this adjustment process (see Figure 9).

For instance, in the spreader trajectory of a loading cycle illustrated in Figure 9a the crane begins lifting the detached spreader from the container on the truck at 0.0 s, reaching its highest position at approximately 9.0 s. The descent starts around 15.2 s, with the spreader halting at the stack entrance at about 25.1 s. At 48.9 s, the spreader resumes its descent into the stack shaft and reaches the container within the stack at approximately 58.0 s (23.8 s after the initial attempt). In another loading cycle depicted in Figure 9b, the operator adjusted the spreader’s descent from 18.9 s to 22.2 s. These delays support the hypothesis that containers positioned above the deck are more vulnerable to damage due to unsuccessful hooking events when operators do not allocate sufficient time and effort for precise adjustments.

To summarise the findings, it is important to highlight that despite the significant impact of container damage on the shipping industry, research focusing on comprehensive container monitoring and maintenance remains limited. Addressing this gap is essential for sustaining the growth of containerisation while reducing financial losses and enhancing cargo safety. To support the demands of a growing global trade network, the shipping industry must prioritise research into damage prevention and adopt modern technologies for monitoring. Containers face various types of damage throughout their lifecycle, particularly during port handling, transit, and exposure to harsh environmental conditions, all of which have considerable effects on global trade. At ports, damage often results from improper handling or inadequate stacking. The surge in container traffic, with some ports managing millions of TEUs annually (e.g., over 47 million TEUs in Shanghai), has led to faster and sometimes careless loading and unloading practices. Improper stacking or mishandling by cranes and forklifts can cause containers to become dented, punctured, or shifted, compromising container integrity and the safety and quality of the cargo within. According to the World Shipping Council, such incidents can lead to container stack collapses, posing further risks of cargo spillage or loss.

**Figure 10:**
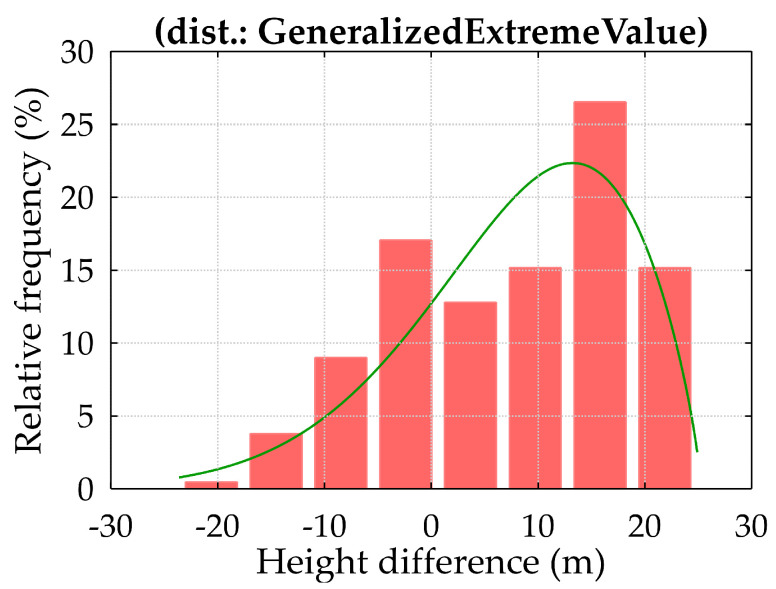
Histogram of spreader lowering heights with best-fit probability distribution function (PDF) representation.

**Figure 11:**
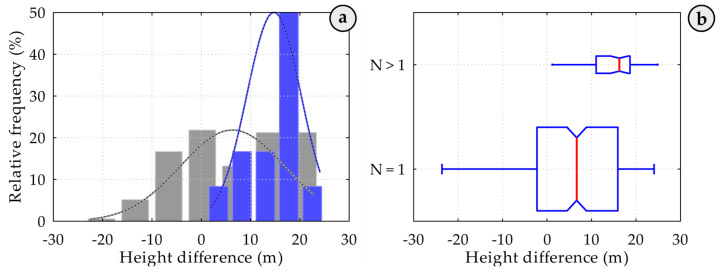
Histogram and probability density functions (PDFs) of spreader lowering heights, where (**a**) illustrates the distribution of first successful container hooking attempts (grey) and repeated attempts (blue), showing that unsuccessful hookings mostly occur at the top of container stacks; while the boxplot in (**b**) compares lowering heights for first (N = 1) and repeated attempts (N > 1), highlighting a lower average height for successful first attempts (6.7 m) and a significantly higher, narrower range for repeated attempts (16.3 m), indicating operator adjustments after initial failures.

The results indicate that unsuccessful hooking attempts primarily occur when containers are positioned above the deck. This pattern is likely due to the limited time operators must fully suppress spreader oscillations in these higher positions, adding complexity to the process. Additionally, the high number of handling procedures each operator must complete per shift may contribute to a decline in operational quality, as the pressure to maintain productivity affects the precision of full-cycle operations. Discussions with company representatives also suggest that certain operational procedures and established practices may contribute to these recurrent errors, highlighting the potential value of reviewing these protocols to identify opportunities for improvement. Supporting these findings, Figure 10 presents a histogram of spreader lowering heights along with the best-fit probability distribution function (PDF) for the data. The analysis shows that containers located at the top of the stack are more frequently unloaded, likely due to the unloading plan designed for a specific day, ship, and crane configuration. This pattern emphasises the impact of unloading schedules on handling operations, indicating that containers positioned for easier access are prioritised, which can influence the frequency and nature of hooking attempts. These findings point to the need for further analysis to optimise handling procedures, reduce unsuccessful hooking operations, and enhance the overall safety and efficiency of container handling operations. Figure 10 displays a histogram of the spreader lowering heights alongside the probability distribution function (PDF) that best fits the data. The results indicate that containers located near the top of the stack were most frequently lifted, likely due to the specific unloading plan tailored for the day, ship, and crane. Figure 11a illustrates the histograms of the differences in spreader lowering heights, accompanied by the best-fit probability distribution functions (PDFs). Heights representing the first successful container hooking are highlighted in grey, while those related to repeated attempts are marked in blue. The data reveal that unsuccessful hooking attempts predominantly occur at the top of container stacks, whereas containers situated below the deck are generally hooked without issue.

The findings from the smaller measurement sample are supported by the height differences, as illustrated in the boxplot presented in Figure 11b. The average descent height of the spreader for a successful first attempt (N = 1) is 6.7 metres, whereas for repeated attempts (N > 1), the average increases significantly to 16.3 metres. This substantial difference suggests that when multiple attempts are necessary, the spreader begins from a higher position, likely due to operator adjustments following prior unsuccessful attempts. Furthermore, the range of descent heights during repeated attempts (N > 1) is noticeably narrower compared to that of successful single attempts (N = 1), indicating more limited operational adjustments when several attempts are required. This variation emphasises the influence of repeated hooking attempts on the efficiency of the handling process and highlights the importance of precision during the initial descent to minimise delays and reduce the risk of container damage. Our observations show that unsuccessful container hooking predominantly occurs atop container stacks above the ship’s deck, whereas containers below the deck are generally secured successfully.

This discrepancy is due to vertical cell guides beneath the deck, which assist in controlling the sway motion of the spreader and container during handling operations. Consequently, it can be inferred that the handling operations on each ship follow a consistent pattern, resulting in similar errors occurring at a relatively stable rate. Feedback from the LKAB “Klaipėdos Smeltė” container terminal confirms that operator actions are standardised, with control procedures generally aligning with established norms. Although the frequency of faulty operations is relatively high, it remains within acceptable operational thresholds. Enhancing the stabilisation of spreaders could improve this situation. The authors acknowledge that the Impact Detection Methodology (IDM) relies on acceleration and vibration sensors, which are inherently susceptible to noise and measurement inaccuracies. These challenges arise from sensor limitations, environmental disturbances, and operational conditions that can introduce unwanted variability in data acquisition. To address these issues, we have implemented robust data filtering techniques, particularly statistical thresholding methods, to differentiate between routine operational vibrations and unintended impact events. High-frequency noise is mitigated using advanced signal processing techniques that enhance clarity in impact detection while preserving critical event data.

Moreover, external environmental factors such as vibrations induced by cranes, wind disturbances, the mechanical tolerances of handling equipment, and surface irregularities can introduce fluctuations in sensor readings. These variations may impact the accuracy of detecting and classifying impact events. To mitigate these effects, our methodology employs adaptive threshold adjustments based on real-time sensor feedback. This adaptive approach dynamically modifies sensitivity levels to accommodate transient disturbances, reducing false positives while ensuring detection reliability. By incorporating these measures, we ensure that our findings accurately reflect real-world handling conditions, thereby enhancing the robustness and applicability of our results.

## 4. Conclusions

This study highlights the challenges associated with container damage during crane-handling operations and proposes the Impact Detection Methodology (IDM) as an effective solution for real-time monitoring and damage mitigation. The findings indicate that unsuccessful hooking attempts predominantly occur when containers are lifted from above-deck positions due to spreader oscillations and high operational workloads. By integrating the IDM with existing crane management technologies and sway control systems, operational precision can be enhanced, thereby reducing damage-related costs and delays.

The results further underscore the necessity for broader experimentation to validate the IDM’s full potential across various port environments. Given the financial and logistical ramifications of container damage, adopting real-time monitoring solutions can enhance operational efficiency, mitigate risks, and contribute to more sustainable global containerisation. Future research should concentrate on refining the IDM’s adaptability, expanding its implementation, and integrating advanced automation for optimal container-handling efficiency. Looking forward, forthcoming research will examine the integration of advanced sway control systems with existing crane control and management frameworks. This integration aims to enhance operational stability and provide real-time feedback to operators, empowering more informed decision-making in specific handling scenarios. Additionally, further investigations will focus on refining the system’s sensitivity to various environmental and unpredictable factors, such as load variations, sudden dynamic shifts, and external mechanical influences. By advancing these aspects, we aim to improve the precision and adaptability of the IDM, ensuring its effectiveness across a wide range of industrial applications.

## Data Availability

Data samples can be provided upon request.
